# A novel TIMM8A mutation in Mohr-Tranebjaerg syndrome without hearing loss and with basal ganglia iron deposition

**DOI:** 10.1186/s13023-025-03868-0

**Published:** 2025-07-01

**Authors:** Ignacio Ventura, Francisco Revert-Ros, Fernando Revert, Jesús Ángel Prieto-Ruiz, José Miguel Hernández-Andreu

**Affiliations:** Grupo de Medicina Molecular y Mitocondrial, Facultad de Medicina y Ciencias de la Salud, Universidad Católica de Valencia San Vicente Mártir, c/Quevedo 2, Valencia, 46001 Spain

**Keywords:** Mohr-Tranebjaerg syndrome, TIMM8A mutation, Dystonia, Iron deposition, Basal ganglia, Mitochondrial dysfunction, Whole-exome sequencing (WES), Neurodegenerative disorders, Susceptibility-weighted imaging (SWI), Iron metabolism dysregulation

## Abstract

Mohr-Tranebjaerg syndrome (MTS) is a rare X-linked recessive neurodegenerative disorder caused by pathogenic variants in the TIMM8A gene. TIMM8A, also known as Deafness-Dystonia Peptide-1 (DDP1) is a mitochondrial intermembrane space protein involved in the import and insertion of hydrophobic membrane proteins from the cytoplasm into the mitochondrial inner membrane. MTS typically presents early-onset progressive hearing loss, dystonia, visual impairment, and cognitive decline. Here, we report a case of a male adolescent with a previously undescribed variant in TIMM8A, associated with progressive dystonia but no hearing loss, highlighting the clinical variability of MTS. A 16-year-old male was referred for genetic evaluation due to a 6-year history of progressive dystonia, motor coordination difficulties, and iron deposits in the basal ganglia detected by brain MRI. Family history revealed mild motor abnormalities in his maternal uncle and recurrent muscle spasms in his mother. Whole-exome sequencing (WES) identified a c.98_101dupAGCA variant in TIMM8A in hemizygosity, classified as likely pathogenic. This variant causes a frameshift leading to a truncated protein. The patient inherited the variant from his mother, who is heterozygous for the mutation. Although the patient lacks the characteristic early-onset hearing loss seen in MTS, his neurological presentation and the imaging findings are consistent with the syndrome. This case underscores the phenotypic heterogeneity of Mohr-Tranebjaerg syndrome, where patients may present with prominent neurological symptoms such as dystonia without the hallmark auditory dysfunction. The identification of a novel TIMM8A variant expands the mutational spectrum of this rare disorder and provides insights into genotype-phenotype correlations. The absence of hearing loss in this patient raises important questions about the variability in the expression of the mutated TIMM8A. This report highlights a novel TIMM8A mutation associated with Mohr-Tranebjaerg syndrome, presenting primarily with dystonia and iron accumulation in the basal ganglia. The findings contribute to the understanding of the clinical spectrum of MTS and emphasize the importance of genetic testing in patients with unexplained progressive neurological symptoms.

## Introduction

Mohr-Tranebjaerg syndrome (MTS), also known as deafness-dystonia-optic neuropathy syndrome, is a rare neurodegenerative disorder linked to mutations in the TIMM8A gene located on the X chromosome [1]. First described in 1960, MTS primarily affects males due to its X-linked recessive inheritance pattern. The disease is characterized by early-onset sensorineural hearing loss, followed by the progressive development of dystonia, ataxia, optic neuropathy, and cognitive decline. These symptoms typically appear during adolescence and early adulthood, significantly impacting quality of life [2]. MTS results from defects in the assembly of the DDP1/TIMM8a-TIMM13 complex, which is essential for proper mitochondrial protein functioning. TIMM8A encodes a protein that is part of the mitochondrial protein import machinery [3]. The syndrome also presents with reduced levels of Tim23 in the inner mitochondrial membrane due to defective assembly of the DDP1/TIMM8a-TIMM13 complex. This syndrome is the result of reduced Tim23 levels in the inner mitochondrial membrane due to defective assembly of the DDP1/TIMM8a-TIMM13 complex [4].

Neuroimaging studies have identified neurodegenerative changes in patients with deletions encompassing the TIMM8A gene [5]. Pathogenic variants in TIMM8A disrupt mitochondrial function, contributing to the multi-systemic nature of the syndrome. Although hearing loss is considered the hallmark of MTS, significant variability exists in the clinical presentation, with some patients exhibiting predominantly neurological symptoms such as dystonia and motor dysfunction.

Interestingly, TIMM8A overexpression correlates with poor prognosis in breast cancer, with patients showing significantly lower overall survival compared to those with low expression. This suggests that TIMM8A could serve as a prognostic biomarker and a potential therapeutic target in breast cancer [6]. Genetic analyses, including next-generation mate-pair sequencing, have been used to identify and characterize deletions in the TIMM8A gene. These studies are crucial for understanding the genetic basis of Mohr-Tranebjaerg syndrome and other related disorders [7].

In this report, we describe a novel TIMM8A variant in a male patient with progressive dystonia, motor coordination difficulties, and iron deposits in the basal ganglia, but notably without hearing impairment. This case underscores the phenotypic heterogeneity of MTS and highlights the importance of genetic testing in patients with atypical presentations of the syndrome and opens the door for additional studies on the possible involvement of TIMM8A in brain iron accumulation.

## Materials and methods

### Clinical evaluation

The patient, a 16-year-old male, was referred to the Genetics Consultation at the Hospital Universitario Araba (HUA) for evaluation of progressive dystonia and motor coordination difficulties, with symptom onset at the age of 10. A detailed family history was obtained, revealing mild motor symptoms in the maternal uncle and recurrent muscle spasms in the patient’s mother. Neurological examination confirmed generalized dystonia, particularly affecting the upper extremities and cervical muscles.

### Genetic analysis

Genomic DNA was extracted from peripheral blood samples using standard procedures. Whole-exome sequencing (WES) was performed on the patient and both parents (trio-based approach) using the Nextera DNA Flex Pre-Enrichment Library Prep and the Illumina Exome Panel on the Illumina NovaSeq 6000 platform. Sequencing data were aligned to the human reference genome (GRCh37), and variant calling was performed using a custom bioinformatics pipeline developed by Igenomix. Variants were filtered and prioritized based on clinical relevance and pathogenicity, using in silico prediction tools such as SIFT, PolyPhen, and MutationTaster, as well as population databases like gnomAD and ClinVar.

### Radiological analysis

A brain magnetic resonance imaging (MRI) was performed on a patient with a diagnosis of progressive generalized dystonia. The examination included axial T2-weighted and axial FLAIR sequences to evaluate the white matter and basal ganglia, using standard imaging parameters (WL: 945–1026, WW: 1643–1784). Images were acquired with a 1.5 Tesla MRI scanner, using high-resolution axial slices. Comparative studies included previous examinations conducted in 2014 and 2019. Specific focus was placed on the globus pallidus, caudate nuclei, and putamen to assess for iron deposits or morphological changes.

### Bioinformatic applications

Protein sequence alignment was performed with the ClustalW application on the EMBL-EBI website, with default settings. Predictions of three-dimensional structure of protein complexes were carried out with AlphaFold Multimer [8] application, accessed via Tamarind Bio website.

## Results

### Clinical findings

The clinical history of the patient, a 16-year-old male, underscores a progressive neurological decline, particularly marked by dystonia that began at the age of 10. His early symptoms involved fine motor difficulties, which evolved into more severe motor impairments, affecting both his upper limbs and neck, and leading to complications such as dysphagia and intermittent myalgias. The neurological examination reveals increased muscle tone and signs of hypertonia, such as ankle clonus. Table 1 provides a detailed overview of the patient’s clinical presentation, imaging findings, and how they correlate with his genetic profile, specifically focusing on the deposition of iron in the basal ganglia and progressive dystonia. The absence of hearing loss emphasizes the phenotypic variability in patients with Mohr-Tranebjaerg syndrome, and the normal biochemical findings rule out other potential diagnoses, such as Wilson’s disease, further reinforcing the link to mitochondrial dysfunction due to the TIMM8A mutation.

**Table 1:** Main clinical symptoms and other findings in the patient with TIMM8A mutation

Clinical Symptom	Other related findings
Progressive Dystonia	Generalized dystonia, especially in upper extremities and neck. Significant motor dysfunction, muscle rigidity
Iron Deposition in Basal Ganglia	MRI showed bilateral hypointensities in the globus pallidus. Susceptibility-weighted imaging (SWI) confirmed iron buildup
Dysphagia	Difficulty swallowing liquids and solids. Gradual worsening, no major improvement
Absence of Hearing Loss	No auditory impairment reported. Normal hearing tests, no signs of auditory neuropathy

### Family history

**Fig. 1:**
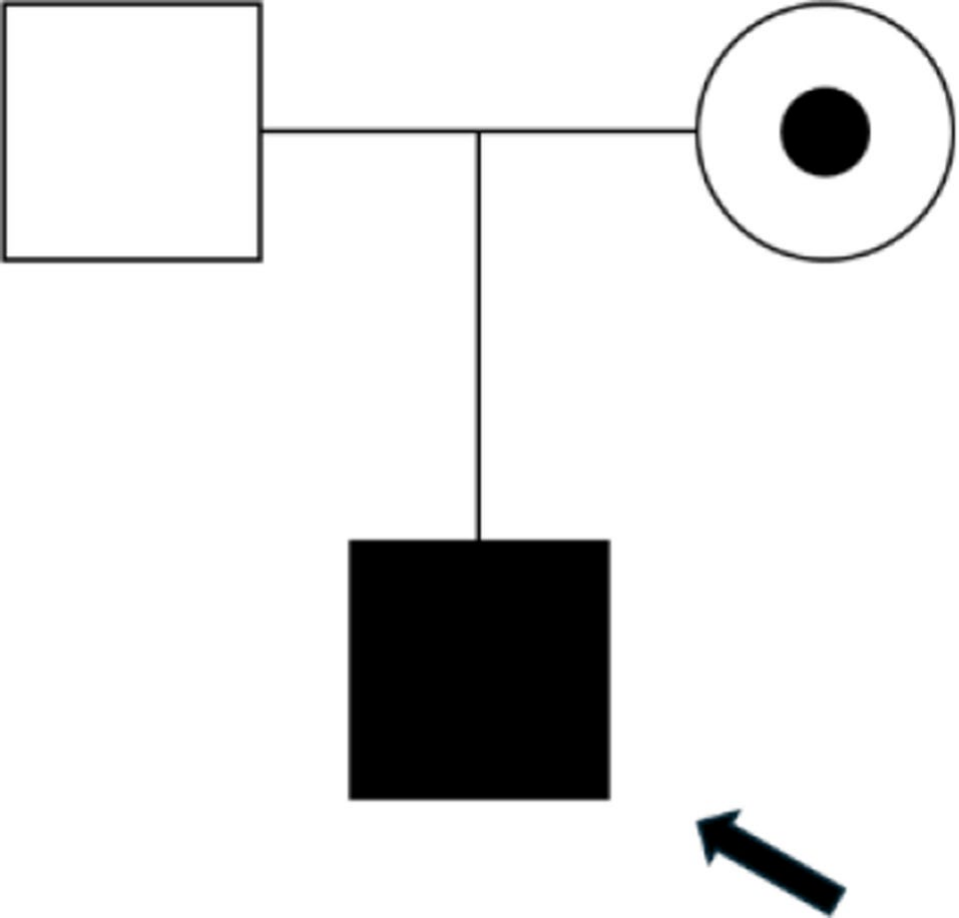
Pedigree of the affected family: Pedigree of the family, showing the proband (indicated by the arrow), a 16-year-old male affected by progressive dystonia. The mother is a carrier of the TIMM8A mutation, represented by a circle with a central dot. The proband is represented by a filled square, indicating an affected male, while the father is unaffected

The patient’s family history reveals a pattern of mild, episodic neuromuscular symptoms, primarily affecting the maternal side. His mother, now 43 years old, reported occasional episodes of muscle spasms and finger stiffness, while her brother experienced similar symptoms during childhood, including motor clumsiness and finger spasms. The uncle’s symptoms improved with age, though he developed persistent facial tics that began in adolescence. Despite the presence of motor symptoms in both the mother and uncle, neither reported hearing loss or visual impairments, which are often associated with Mohr-Tranebjaerg syndrome. Figure 1 represents the family history, highlighting potential genetic transmission patterns and phenotypic variability.

**Fig. 2:**
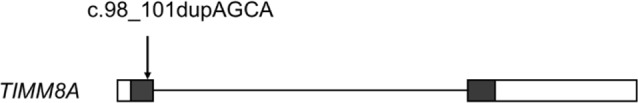
Schematic representation of the TIMM8A gene, showing the identified c.98_101dupAGCA mutation. This mutation is in a critical region of the gene associated with mitochondrial dysfunction, which is characteristic of Mohr-Tranebjaerg syndrome

### Genetic findings

Whole-exome sequencing (WES) performed on the patient and his parents identified a novel hemizygous variant in the TIMM8A gene (c.98_101dupAGCA; p.Leu35fs) located on the X chromosome (Table 1). This variant causes a frameshift within exon 1 and a premature stop codon leading to a truncated protein (Fig. 2). The patient’s mother was confirmed to be a heterozygous carrier of the same variant. Bioinformatic analysis predicted this variant to be likely pathogenic, based on in silico prediction tools (SIFT, PolyPhen, MutationTaster) and population databases (gnomAD). No other significant variants were found in genes associated with dystonia or iron metabolism (Table 2).

**Table 2:** Genetic test results for TIMM8A mutation in the patient and carrier mother: the TIMM8A gene mutation (c.98_101dupAGCA; p.(Leu35fs)) is confirmed as likely pathogenic, with the patient being hemizygous and the mother being heterozygous carrier. Sanger sequencing was used to confirm the carrier status in the mother

Gene	Variant	Mutation Type	Inheritance	Clinical Significance	Confirmation Method
TIMM8A	c.98_101dupAGCA; p.(Leu35fs)	Frameshift	X-linked recessive	Likely pathogenic	Whole-exome sequencing (WES)
Mother (Carrier)	c.98_101dupAGCA; p.(Leu35fs)	Heterozygous	X-linked	Carrier	Sanger sequencing

### Variant confirmation and classification

The identified variant in TIMM8A (c.98_101dupAGCA; p.Leu35fs) was confirmed by Sanger sequencing in both the patient and the mother, who was found to be a heterozygous carrier. The variant was classified as “likely pathogenic” according to the American College of Medical Genetics (ACMG) guidelines (Table 3).

**Table 3:** TIMM8A gene variants associated with deafness dystonia syndrome: this table summarizes the TIMM8A gene variants that have been described in the NCBI database and are associated with deafness dystonia syndrome. These variants involve both insertions and deletions, leading to frameshift mutations. Such mutations result in the production of truncated or non-functional proteins, which contribute to the pathophysiology of the syndrome. The classification of each variant as either pathogenic or likely pathogenic is based on current genetic evaluations in the database. The review status “G” indicates that these variants have undergone confirmed genetic review

Variation	Gene (Protein Change)	Type (Consequence)	Condition	Classification	Review Status
NM_004085.4(TIMM8A) .232_233insCAAT	TIMM8A (p.Leu78fs)	Insertion (3’ UTR variant + 1 more)	Deafness dystonia syndrome	Likely pathogenic	G
NM_004085.4(TIMM8A) .181del	TIMM8A (p.Ala61fs)	Deletion (3’ UTR variant + 1 more)	Deafness dystonia syndrome	Pathogenic	G
NM_004085.4(TIMM8A) .148_157del	TIMM8A (p.Lys50fs)	Deletion (3’ UTR variant + 1 more)	Deafness dystonia syndrome	Pathogenic	**G**
NM_004085.4(TIMM8A) .127del	TIMM8A (p.Cys43fs)	Deletion (frameshift variant)	Deafness dystonia syndrome	Pathogenic	G
NM_004085.4(TIMM8A) .116del	TIMM8A (p.Met39fs)	Deletion (frameshift variant)	Deafness dystonia syndrome	Pathogenic	G
NM_004085.4(TIMM8A) .66_67del	TIMM8A (p.Ile23fs)	Deletion (frameshift variant)	Deafness dystonia syndrome	Pathogenic	**G**

**Fig. 3:**
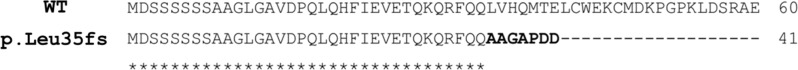
Alignment of the sixty first amino acids of the sequence of Wild Type (WT) TIMM8A protein with that of p.Leu35fs mutant

The p.Leu35fs mutation found in our patient results in a significant alteration of the TIMM8A protein structure, as depicted in Fig. 3. This mutation causes a frameshift starting at amino acid 35, leading to the incorporation of an aberrant peptide sequence (AAGAPDD) and subsequent truncation of the protein. The alignment between the wild-type and mutant TIMM8A protein sequences highlights the extent of this disruption, where the mutant form shows substantial deviation from the normal sequence after the frameshift.

**Fig. 4:**
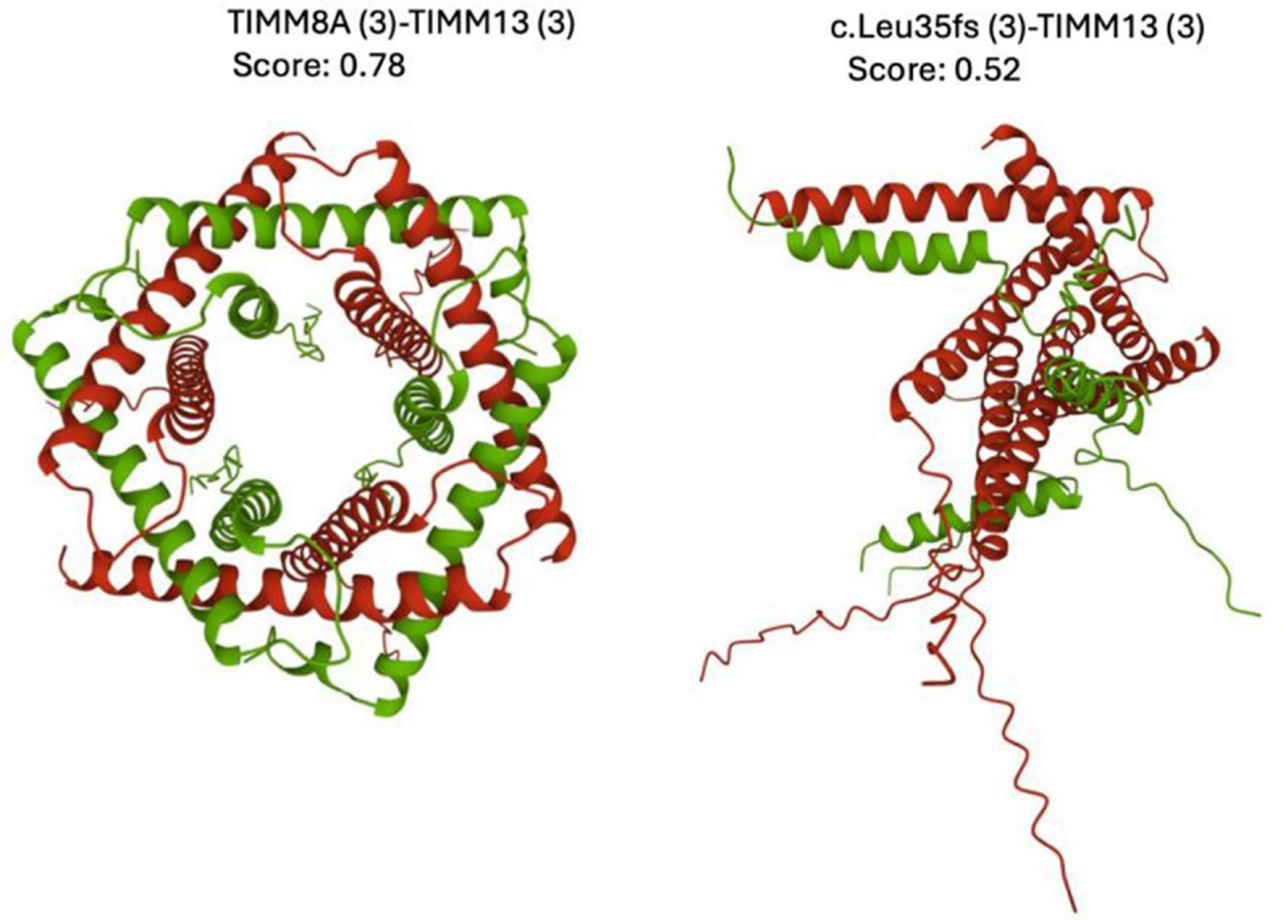
Effect of the c.98_101dupAGCA mutation in TIMM8A on the TIMM8A-TIMM13 complex assembly. The human wild type TIMM8A (green) and TIMM13 (red) polypeptides are predicted to form a structured heterohexamer with a 3:3 stoichiometry, according to AlphaFold Multimer software (left). The TIMM8A p.Leu35fs mutant (green) is predicted to completely disrupt the hexamer (right), highlighting the critical role of TIMM8A in mitochondrial function

Figure 4 illustrates the expected impact of the TIMM8A gene c.98_101dupAGCA mutation on the structural integrity of the TIMM8A-TIMM13 complex, according to AlphaFold Multimer software predictions. On the left, human wild-type TIMM8A (green) and TIMM13 (red) proteins are predicted to form a well-organized heterohexamer with a 3:3 stoichiometry. This structure is like the experimentally solved for the TIM8-TIM13 complex from Saccharomyces cerevisiae [https://www.rcsb.org/structure/3CJH]. In contrast, AlphaFold Multimer does not predict TIMM8A p.Leu35fs mutant (green) to form an organized hexamer with wild-type TIMM13 (red), but a disordered oligomeric structure. Although the confidence of the oligomeric structure predicted for p.Leu35fs is low (score: 0.52), the expected loss of an ordered hexameric organization compromises the import and maintenance of mitochondrial proteins.

During the genetic analysis of the patient, several variants of potential clinical relevance were identified. Although their direct involvement in the patient’s primary clinical presentation could not be fully confirmed, these variants are noteworthy due to their partial association with the clinical indication and their presence in genes that play roles in cellular processes potentially relevant to the disease. Some of these genes are currently not well characterized, and the pathogenic significance of these variants remains uncertain due to a lack of direct evidence or the mode of inheritance.

Table 4 summarizes the additional variants detected, highlighting key genomic details such as their chromosomal position, zygosity, and predicted pathogenicity. The analysis revealed variants with a spectrum of potential impacts, ranging from low to moderate pathogenic predictions based on computational tools. These variants include changes in genes like MARVELD3, SMG1, and SOWAHD, which have been associated with cellular functions related to mitochondrial integrity and gene regulation. However, due to the current limitations in genetic interpretation, these findings are categorized as variants of uncertain significance (VUS). Further research is needed to elucidate the potential role of these variants in the patient’s clinical presentation.

**Table 4:** Genetic variants with potential clinical relevance detected in the patient’s genetic analysis: this table presents additional genetic variants identified during the patient’s genetic analysis. These variants, found in genes such as MARVELD3, SMG1, and SOWAHD, are categorized as variants of uncertain significance (VUS) based on current genetic evaluation guidelines. Each entry includes relevant genomic informationand pathogenicity predictions based on computational tools like the CADD scores. The presence of these variants highlights the complexity of genetic interpretation in rare diseases and underscores the need for further studies to assess their potential clinical impact

Gene	OMIM	cDNA Change	Protein Change	Effect	SNP ID	Zygosity	Pathogenicity (CADD scores)
MARVELD3	614,094	c.253 C > A	p.(Pro85Thr)	Missense	rs201459139	Heterozygous	Low
SMG1	607,032	c.802 C > T	p.(Pro268Ser)	Missense	rs138794099	Heterozigous	Moderate
SMG1	607,032	c.9392 A > G	p.(Lys3131Arg)	Missense	rs751727166	Heterozigous	Moderate
SMG1	607,032	c.34_36delAGC	p.(Ser12del)	Inframe Deletion	rs773997323	Heterozigous	Moderate
SOWAHD	97%	c.286_287delGA	p.(Glu96Met)	Missense	No data	Hemizygous	Low

### Radiological findings

**Fig. 5:**
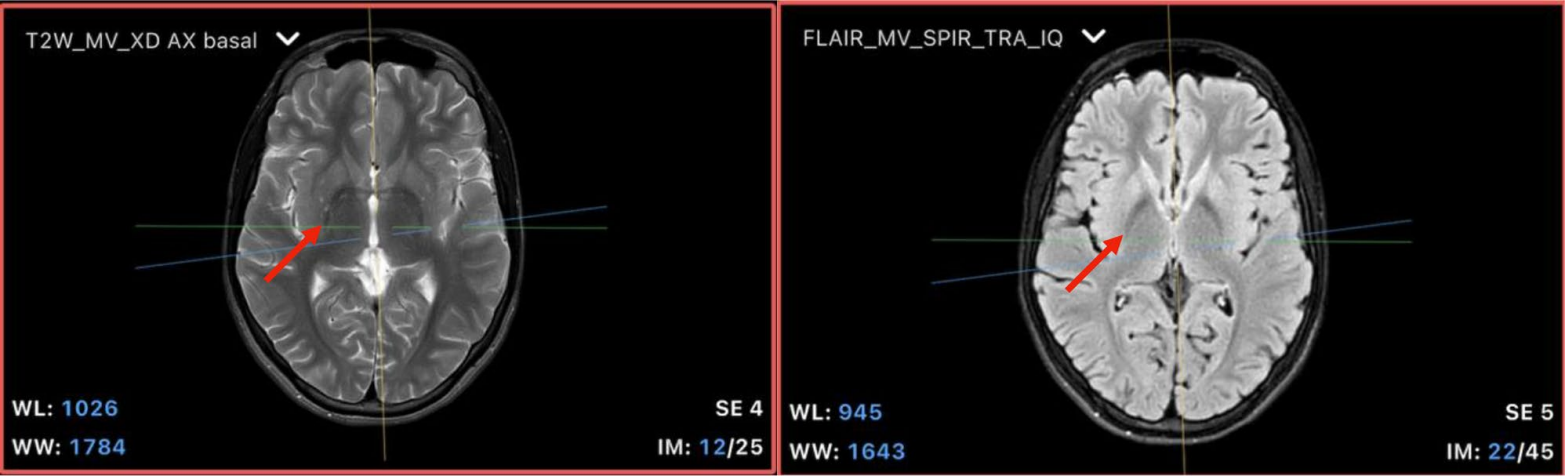
Axial MRI showing iron deposition in the globus pallidus: MRI showing bilateral iron deposition in the globus pallidus, highlighted by susceptibility-weighted imaging (SWI). Left: Axial T2-weighted sequence showing bilateral hypointensity in the globus pallidus (red arrow), suggestive of iron deposition. Right: Axial FLAIR sequence confirming the magnetic susceptibility alteration in the globus pallidus (red arrow), with preservation of the internal medullary lamina

Brain magnetic resonance imaging (MRI) conducted in 2014 and 2019 showed bilateral hypointensities in the basal ganglia, specifically in the globus pallidus, suggesting iron deposits. These findings were identified in susceptibility-weighted imaging (SWI) and T2*-weighted sequences. No other abnormalities were observed in other parts of the basal ganglia, and there was no cerebellar atrophy or brainstem involvement. Spectroscopy results showed normal metabolite ratios with no significant changes compared to previous studies. Additionally, there were no abnormalities in the supratentorial or infratentorial white matter. The ventricular system and cortical sulci were within normal limits for the patient’s age, ruling out hydrocephalus or cortical atrophy (Fig. 5).

### Biochemical findings

Despite the presence of iron deposits in the brain, blood tests showed normal levels of ferritin (77 ng/mL), transferrin (185 mg/dL), and a transferrin saturation index of 22%. Serum ceruloplasmin and copper levels were within normal limits, although urinary copper levels were slightly elevated (5 mcg/dL). Fecal calprotectin tests were negative, and stool parasitology and cultures showed no infections. No abnormalities were detected in liver function, renal function, or thyroid hormone levels. Genetic tests for PKAN syndrome and other disorders related to iron metabolism (ATP13A2, C19orf12, PANK2) were negative.

### Therapeutic interventions

Given the progression of dystonia, the patient was started on Levodopa/Carbidopaat 25/100 mg three times a day, but no significant clinical improvement was observed. The patient also initiated experimental treatment with Pantothenic acid and Pantethine. This treatment was hypothesized to reduce intracellular iron accumulation. The clinical effects of this therapy are still under evaluation (Table 5).

**Table 5:** Treatments and recommendations based on genetic and clinical findings

Aspect	Details	Recommendations	Treatment Status
Genetic Diagnosis	TIMM8A mutation (c.98_101dupAGCA; p.(Leu35fs))	Genetic counseling for the family, including potential future testing	Confirmed through WES and Sanger sequencing
Neurological Symptoms	Progressive dystonia, motor dysfunction, dysphagia	Physical therapy for motor skills, speech therapy for dysphagia	Ongoing
Iron Accumulation (MRI findings)	Iron deposition in the basal ganglia (globus pallidus)	Regular monitoring of iron metabolism and MRI scans	Under observation
Absence of Hearing Loss	No auditory impairment	Regular hearing tests to monitor any potential onset of hearing loss	No symptoms
Biochemical Findings	Normal ferritin, transferrin, and copper levels	No intervention needed for copper metabolism disorders	No treatment required
Prenatal Diagnosis	Mother is a carrier of the TIMM8A mutation	Carrier screening for potential future pregnancies	Recommended for future pregnancies
Experimental Therapies	None currently implemented	**Consideration for iron-chelation therapy or mitochondrial-targeted treatments**	Under evaluation

## Discussion

The absence of hearing loss in our patient, which is typically an early and defining feature of MTS, underscores the phenotypic variability of the syndrome. While hearing loss is often the first symptom of the disease, its absence in this case, alongside the presence of iron deposition, suggests that different TIMM8A mutations may lead to distinct clinical manifestations. This observation highlights the genotype-phenotype correlation in MTS and suggests that certain mutations may predispose patients to a motor phenotype rather than the classic auditory and optic neuropathies.

The c.98_101dupAGCA mutation present in our patient is expected to produce a truncated polypeptide (p.Leu35fs) derived from a frameshift and premature stop codon (PSC) within exon 1 of the TIMM8A gene. PSCs arising within non-3’-terminal exons typically lead to nonsense-mediated mRNA decay (NMD) provided the PSC is at least 50–55 nucleotides upstream of an exon-exon junction [9]. In the case of the c.98_101dupAGCA mutation, the PSC is only 10 nucleotides upstream of the exon 1:exon 2 junction, so the mutant mRNA is not expected to undergo NMD and its translation into the TIMM8A p.Leu35fs is likely to be effective. Furthermore, this patient’s unique set of symptoms probably indicates that the disease is not a mere consequence of the complete absence of the TIMM8A protein but is due to specific properties of the TIMM8A p.Leu35fs mutant. The finding of iron accumulation in the basal ganglia of this patient suggests a potential link between the mitochondrial dysfunction induced by this mutation and the disruption of mitochondrial iron regulation, like in other neurodegenerative diseases associated with iron metabolism disorders (NBIAs), such as X-linked cerebellar ataxia and sideroblastic anemia [10]. The accumulation of iron in the brain is also emerging as a significant factor in the progression of other neurodegenerative disorders, such as sclerosis multiple [11]. Since our patient shows basal ganglia iron deposition, which is an unusual characteristic of MTS, we try to provide a broader perspective on the genetic and clinical features of Mohr-Tranebjaerg syndrome with Table 6 which offers a comparative overview of this disorder in relation to other mitochondrial diseases that also exhibit iron metabolism dysfunction and neurological symptoms. These disorders, while sharing common themes of mitochondrial dysfunction and abnormal iron handling, present distinct clinical manifestations and genetic mutations. For example, Mohr-Tranebjaerg syndrome (linked to TIMM8A mutations) is marked by severe neurodegenerative symptoms, particularly dystonia and motor dysfunction, accompanied by iron accumulation in the basal ganglia. In contrast, XLSA and XLSA/A (both associated with mitochondrial dysfunction) display different features, such as ataxia in XLSA/A and anemia in both. Friedreich Ataxia (caused by mutations in the FXN gene) presents a broader dysregulation of mitochondrial iron across various tissues, including the brain, heart, and skeletal muscle. Additionally, HML highlights another mitochondrial disorder involving iron buildup, but primarily affecting skeletal muscle. This comparative table enables a clearer understanding of how mitochondrial dysfunction involving iron accumulation can lead to a spectrum of neurological and systemic symptoms across different genetic contexts (Table 6).

**Table 6:** Genetic variants and clinical features in Mohr-Tranebjaerg syndrome vs. other mitochondrial disorders with Iron accumulation

Disorder	Gene	Mutation	Clinical Features	Iron Metabolism Impact	Neuroimaging Findings
Mohr-Tranebjaerg Syndrome (MTS)	TIMM8A	c.98_101dupAGCA; p.(Leu35fs)	Progressive dystonia, motor dysfunction, dysphagia, absence of hearing loss	Disruption of mitochondrial function, iron accumulation	Iron deposits in basal ganglia (globus pallidus)
X-Linked Sideroblastic Anemia (XLSA)	ALAS2	Mutations in ALAS2	Hypochromic microcytic anemia, iron overload in erythroblasts	Impaired heme synthesis, mitochondrial iron sequestration	Iron accumulation in erythroblasts
X-Linked Sideroblastic Anemia with Ataxia (XLSA/A)	ABCB7	Mutations in ABCB7	Mild sideroblastic anemia, early-onset non-progressive cerebellar ataxia	Mitochondrial iron overload, impaired ISC export	Iron accumulation in erythroblasts, no parenchymal iron deposition
Frataxine Deficiency (Friedreich Ataxia)	FXN	Expansions in FXN gene (GAA repeats)	Ataxia, cardiomyopathy, skeletal deformities, diabetes	Iron dysregulation in mitochondria, impaired iron metabolism	Brainstem and spinal cord iron accumulation
Hereditary Myopathy with Lactic Acidosis (HML)	ISCU	Mutation in ISCU (intronic)	Exercise intolerance, myoglobinuria, lactic acidosis	Mitochondrial iron accumulation, decreased ISC proteins	Mitochondrial iron deposition in skeletal muscle

These findings emphasize the complexity of genetic data interpretation, particularly when dealing with rare diseases and novel genetic mutations. The inclusion of these variants highlights the importance of ongoing genetic evaluation and the potential need for additional functional studies to clarify their clinical relevance.

In a recent publication, Araújo Salomão et al. (2024), when studying a group of Brazilian patients with neurodegeneration with brain iron accumulation, identified a patient with a mutation in TIMM8A (c.127T > C, p.Cys43Arg) showing bilateral symmetric iron deposits in globus pallidus and substantia nigra [12]. This patient showed cognitive decline and generalized dystonia including tremor, bradykinesia, postural instability and dysphagia. In contrast with our findings, deafness was a present in this patient, but no co-segregation studies were available and a exome sequencing was not performed for this patient so additional mutations could not be discarded. However, to our knowledge, iron deposition in the globus pallidus has not been widely studied and reported in patients with Mohr-Tranebjaerg syndrome. Our patient exhibits bilateral iron deposits in the globus pallidus, as evidenced by susceptibility-weighted imaging (SWI) on MRI, in conjunction with progressive dystonia. This finding may represent a novel aspect of the disease spectrum in MTS, raising important questions about the potential role of iron metabolism in the pathophysiology of this rare syndrome. Iron deposition in neurodegenerative diseases often reflects underlying mitochondrial dysfunction, oxidative stress, or abnormalities in iron homeostasis. Given that TIMM8A encodes a mitochondrial translocase protein involved in the import of polypeptides into the mitochondrial intermembrane space, it is plausible that disruptions in mitochondrial function, caused by the loss of TIMM8A function, may contribute to the impaired regulation of iron metabolism. This could result in the abnormal accumulation of iron within the basal ganglia, like what is observed in other mitochondrial disorders. The identification of iron deposition in this patient supports the hypothesis that mitochondrial dysfunction may play a key role in the neurodegenerative process observed in MTS. The Ferritin, transferrin, and transferrin saturation levels are normal, but the iron accumulation in the basal ganglia is a relevant finding, suggesting that despite normal blood levels, there might be an iron metabolism disorder at the mitochondrial level. The slightly elevated urinary copper might indicate a potential imbalance in copper metabolism, though it is not clinically significant in this context. Ceruloplasmin and serum copper levels are normal, ruling out copper metabolism diseases like Wilson’s disease.

This case, therefore, suggests that iron accumulation could be a previously underrecognized feature of MTS, particularly in patients who present with predominant neurological symptoms such as dystonia. It is possible that iron deposition contributes to the progressive neurodegeneration seen in these patients, as the basal ganglia is critical for motor control, and iron dysregulation in these regions could exacerbate motor dysfunction.

**Fig. 6:**
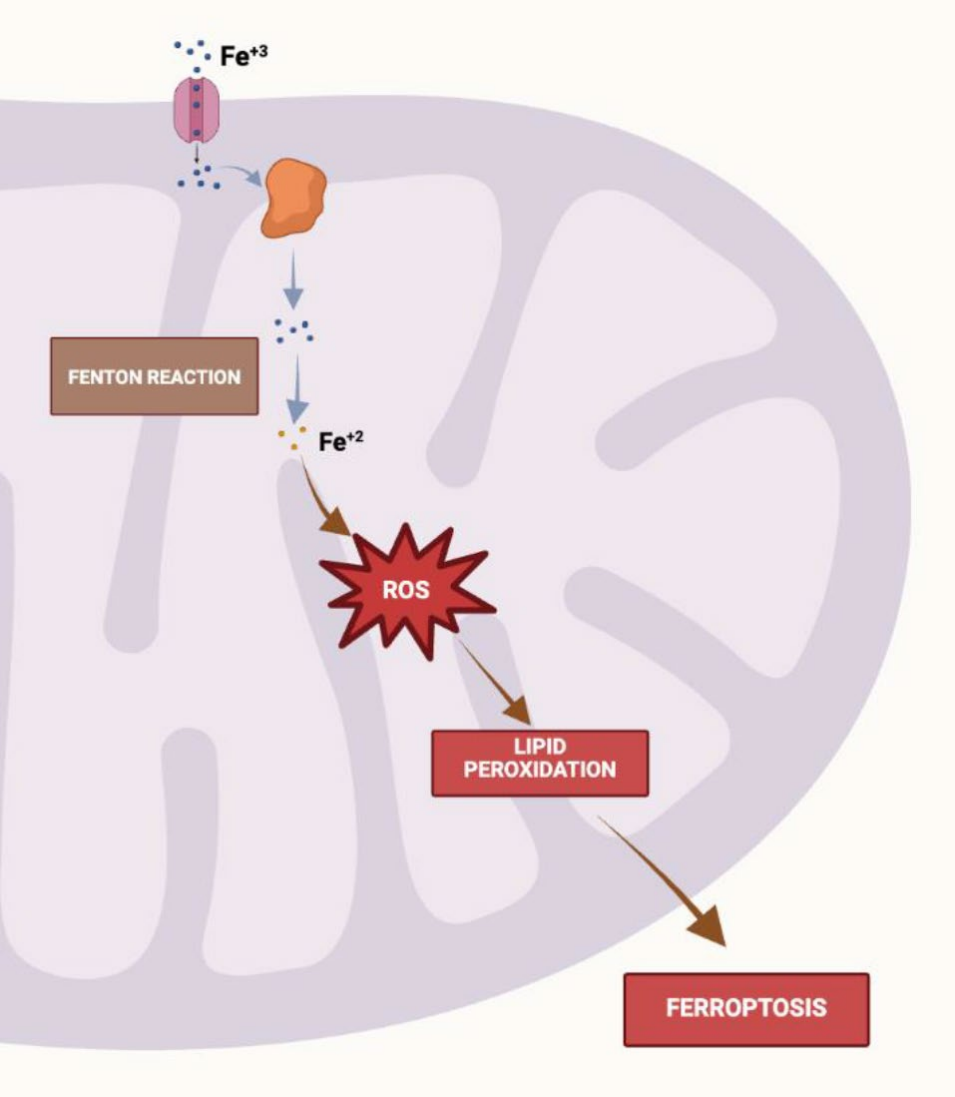
Process of ferroptosis. Mitochondrial dysfunction, iron dysregulation, and their connection to cell death mechanisms. The figure illustrates how overloading of the mitochondrial iron pool triggers the Fenton reaction, producing mitochondrial reactive oxygen species (ROS). The reaction of ROS with unsaturated fatty acids in the mitochondrial membrane generates lipid peroxidation, inducing ferroptosis. (Image created with BioRender)

Ferroptosis is a regulated form of cell death driven by iron-dependent lipid peroxidation, which leads to membrane damage and eventual cell death. Neurons, particularly in iron-rich areas like the basal ganglia, are highly susceptible to this process due to their high content of polyunsaturated fatty acids and the potential for oxidative stress. In this case, the dysregulation of iron homeostasis caused by mitochondrial dysfunction may exacerbate oxidative damage, promoting ferroptosis and contributing to the progressive motor dysfunction and dystonia observed in the patient. Therefore, exploring therapeutic strategies that target ferroptosis, such as iron chelation or antioxidants, could represent a novel approach for managing the neurological symptoms associated with this syndrome [13]. Figure 6 illustrates the process of ferroptosis, a form of programmed cell death driven by iron accumulation, mitochondrial dysfunction, and oxidative stress. The pathway begins with the uptake of iron through the transferrin-transferrin receptor complex, leading to the conversion of ferric iron (Fe³⁺) to ferrous iron (Fe²⁺). This free iron acts as a catalyst for lipid peroxidation, a crucial event in triggering ferroptosis. Mitochondrial dysfunction is also a central factor, disrupting cellular homeostasis by impairing fatty acid oxidation, depleting ATP levels, and increasing reactive oxygen species (ROS). These disturbances cause mitochondrial damage, including protein and lipid oxidation, inflammation, and the opening of the mitochondrial permeability transition pore, leading to cell death. Furthermore, imbalances in mitochondrial dynamics, particularly in fusion and fission processes, exacerbate mitochondrial dysfunction and promote the progression of ferroptosis. This highlights the interconnected roles of iron metabolism and mitochondrial biogenesis in the regulation of cell survival and death [14].

The presence of iron accumulation in the basal ganglia in this case also raises the question of whether therapeutic interventions targeting iron dysregulation, such as iron chelation or mitochondrial therapies, could benefit patients with MTS. Although currently speculative, this hypothesis warrants further investigation, as treatments aimed at reducing iron levels have shown promise in other neurodegenerative disorders involving iron overload [15].

Recent studies have highlighted the role of iron metabolism in neurodegenerative processes, particularly through the overexpression of HO-1 in microglia, leading to neurotoxic iron accumulation and ferroptosis. Fernández-Mendívil et al. (2020) demonstrated how HO-1 overexpression in aging contributes to iron deposits and neurodegeneration, which were effectively mitigated by treatment with the iron chelator Deferoxamine (DFX). Given the observed iron accumulation in the basal ganglia of our patient, the use of Deferoxamine may be a promising therapeutic strategy to reduce iron-related oxidative damage and slow neurodegeneration [16].

Deferoxamine binds to excess iron, forming a complex that can be excreted through the urine, thereby reducing iron overload in tissues [17]. While the use of iron chelation therapy is not a standard treatment for Mohr-Tranebjaerg syndrome (MTS), its potential benefits in cases of mitochondrial iron dysregulation warrant consideration. Regular monitoring of iron levels, along with periodic MRI scans, would be necessary to assess the efficacy of the chelation therapy and to adjust the dose as needed. Additionally, given that deferoxamine is typically administered subcutaneously or intravenously, the treatment regimen should be carefully planned to ensure patient compliance and minimize potential side effects, such as auditory or ocular toxicity, which could be particularly relevant in patients with mitochondrial dysfunction.

## Conclusion

Here we report a novel TIMM8A mutation (c.98_101dupAGCA; p.Leu35fs) associated with Mohr-Tranebjaerg syndrome (MTS), presenting with progressive dystonia and iron accumulation in the basal ganglia. The absence of hearing loss in this patient, a hallmark feature of MTS, underscores the phenotypic variability of the syndrome and suggests that certain mutations may lead to a motor presentation. The identification of iron deposition in the globus pallidus represents a novel finding in MTS and suggests a potential link between mitochondrial dysfunction caused by TIMM8A mutations and abnormalities in iron metabolism.

We propose the use of brain MRI with susceptibility-weighted imaging (SWI) as part of the routine diagnostic workup for patients with dystonia of unknown origin. Additionally, our findings raise the possibility that therapeutic interventions targeting iron dysregulation could benefit patients with MTS. Finally, in our opinion, this case contributes to a better understanding of the genotype-phenotype correlations in MTS and suggests that iron accumulation may play a significant role in the disease’s pathophysiology. Early identification of these features could have important diagnostic and therapeutic implications for patients with TIMM8A-related disorders.

## Data Availability

The data are available by request.
